# Mammalian tracheal development and reconstruction: insights from *in vivo* and *in vitro* studies

**DOI:** 10.1242/dev.198192

**Published:** 2021-07-06

**Authors:** Keishi Kishimoto, Mitsuru Morimoto

**Affiliations:** 1 Laboratory for Lung Development and Regeneration, RIKEN Center for Biosystems Dynamics Research (BDR), Kobe 650-0047, Japan; 2 RIKEN BDR–CuSTOM Joint Laboratory, Cincinnati Children's Hospital Medical Center, Cincinnati, OH 45229, USA; 3 Center for Stem Cell & Organoid Medicine (CuSTOM), Perinatal Institute, Division of Developmental Biology, Cincinnati Children's Hospital Medical Center, Cincinnati, OH 45229, USA; 4 Division of Developmental Biology, Cincinnati Children's Hospital Medical Center, Cincinnati, OH 45229, USA

**Keywords:** Airway, Cartilage, Morphogenesis, Pluripotent cells, Respiratory system, Tubulogenesis

## Abstract

The trachea delivers inhaled air into the lungs for gas exchange. Anomalies in tracheal development can result in life-threatening malformations, such as tracheoesophageal fistula and tracheomalacia. Given the limitations of current therapeutic approaches, development of technologies for the reconstitution of a three-dimensional trachea from stem cells is urgently required. Recently, single-cell sequencing technologies and quantitative analyses from cell to tissue scale have been employed to decipher the cellular basis of tracheal morphogenesis. In this Review, recent advances in mammalian tracheal development and the generation of tracheal tissues from pluripotent stem cells are summarized.

## Abstract (precis)

**Summary:** This Review summarizes the current knowledge of mammalian tracheal development, which is distinct from lung in large part, and discusses the generation of tracheal tissues from human pluripotent stem cells.

## Introduction

**Fig. 1.**
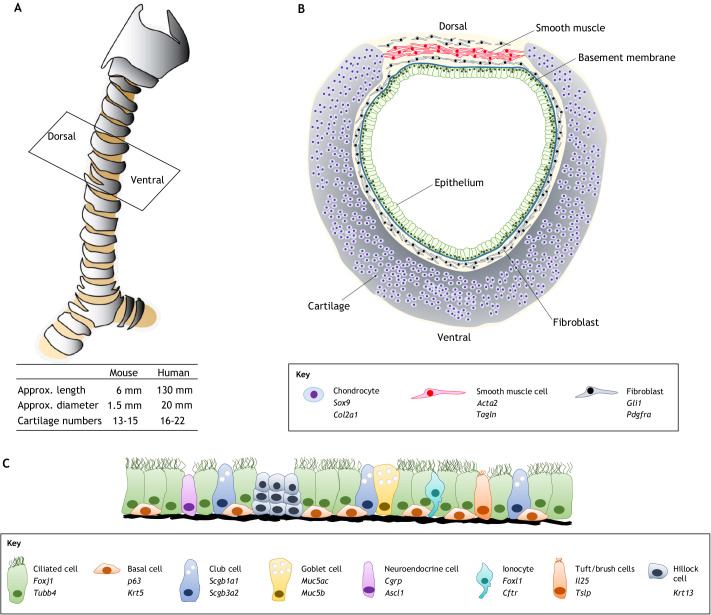
**The structure of the trachea.** (A) An overview of the trachea. The trachea is a cartilaginous straight tubular organ that connects the larynx to the bronchi. (B) A transverse view of mouse trachea, at the position indicated in A. Ventral-lateral crescent-shaped cartilaginous rings and dorsal smooth muscle support the airway structure. (C) Pseudostratified epithelium in the adult trachea. The epithelium consists of ciliated, basal, club, goblet, neuroendocrine, tuft/brush, and hillock cells and ionocytes. Markers of each cell type are listed in the key. *Cgrp* (*Calca*); *p63* (*Trp63*).

The mammalian respiratory system is crucial for postnatal survival. Hence, developmental defects in the respiratory system can result in life-threatening breathing disorders at birth (Morrisey and Hogan, 2010). The trachea is a large tubular air pathway that connects the larynx to the bronchi to deliver external air to the lungs. As the width and luminal smoothness of the trachea determine ventilation efficiency, the development of large tube morphology with smooth epithelia is very important for survival. The length and internal diameter of the human and mouse trachea are approximately 130×20 mm and 6×1.5 mm, respectively (Herbert, 1999; Standring et al., 2005) (Fig. 1A). The trachea has been extensively studied using a mouse model as basic tracheal structure and function are conserved between mice and humans, although certain important differences have also been observed between the two organisms (Box 1) (Conway et al., 2020; Hogan et al., 2014; Morrisey and Hogan, 2010; Rock et al., 2010).

**Box 1. Different and common tracheal features in humans and mice**

Several important differences exist between the human and murine trachea in size and structure. Both the human and murine tracheal epithelium shows pseudostratified epithelium consisting of major airway cell types (i.e. ciliated, club and basal cells), although human tracheal cells are taller than those of mice (Hogan et al., 2014). The cartilage and basal cells are only present in the trachea and the main branch in mice but these components expand to bronchioles in humans. The diameter of the mouse trachea (−1.5 mm) is equivalent to that of the human bronchioles. In addition, the distribution of goblet cells and SMGs is different between mice and humans. Goblet cells are more abundant in the human trachea. Murine SMGs are restricted to the uppermost region of the airway whereas human SMGs can be found extensively throughout the intrapulmonary airways. These insights imply that the mouse trachea resembles human bronchioles, small sections of the airway (Rock et al., 2010).

The epithelium covers the luminal surface to protect respiratory tissues via mucociliary clearance, which entraps inhaled particles and pathogens with mucous that are then moved anteriorly toward the oropharynx by motile cilia (Fig. 1B,C). The tracheal epithelium has a pseudostratified epithelial structure that is composed of a diversity of cell types, including ciliated, secretory club, goblet, neuroendocrine and basal cells, as well as hillock cells and ionocytes (Hogan et al., 2014; Montoro et al., 2018) (Fig. 1C). These cells, except for hillock cells, are also present in the interpulmonary epithelium. It remains to be determined whether these tracheal and interpulmonary epithelial cells are functionally equivalent or partially different. Ciliated cells, which are the predominant cell population covering the lumen (50-80%), drive mucociliary clearance by propelling mucus anteriorly. Basal cells account for ∼30% of the pseudostratified mucociliary epithelium of the trachea and have a flattened shape at the base of the epithelium. Basal cells are known to be stem cells that can undergo self-renewal and differentiate into multiple epithelial cell types (Hong et al., 2004; Rock et al., 2009). Club cells are secretory cells, also known as facultative progenitors, which are slow-cycling in steady state but highly proliferative in response to injury and can transdifferentiate into ciliated and basal cells (Rawlins et al., 2009; Tata et al., 2013). Goblet cells are also secretory cells that secrete mucins, including Muc5ac and Muc5b; these secreted mucins coat the surface of the epithelium and respond to inflammation. Additionally, ionocytes and tuft/brush cells are rare cell types that were more recently identified in human and mouse by single-cell transcriptome analysis (Montoro et al., 2018; Plasschaert et al., 2018). Ionocytes are enriched in ion transport genes and express cystic fibrosis transmembrane conductance regulator (*Cftr*), which, when mutated, leads to cystic fibrosis in humans. Tuft/brush cells are classified as solitary chemosensory cells that respond to neurogenic and immune signals (Krasteva et al., 2011). In addition to these surface epithelial cell types, the submucosal gland (SMG) includes luminal cells and myoepithelial basal cells. The SMG is a gland that secretes mucins and other factors to the surface epithelium. Interestingly, myoepithelial basal cells act as a reserve stem cell population, capable of responding to injury and giving rise to luminal and surface epithelial cells (Liu and Engelhardt, 2008; Tata et al., 2018).

The tracheal mesenchyme consists of tissues, including fibroblasts, crescent-shaped cartilage and trachealis smooth muscle (SM), that balance the rigidity and elasticity of the airway to prevent collapse (Fig. 1B). These mesenchymal populations are involved in airway epithelial maintenance. Some of the subepithelial fibroblasts express growth factors, such as *Fgf10* and *Il6*, to maintain surface epithelium (Hou et al., 2019; Tadokoro et al., 2014). In contrast, periodically patterned cartilaginous rings ensure rigidity, and SM provides elasticity for flexibility. Developmental anomalies of these tissues result in life-threatening congenital malformations, such as tracheostenosis, tracheal agenesis, and tracheomalacia (Box 2) (Schweiger et al., 2016). The tracheal mesenchyme also contains vasculature and intrinsic neurons, which provide oxygen and nutrients and control airway constriction, respectively (Sparrow et al., 1994; Young et al., 2020).

**Box 2. Human congenital tracheal disorders**

Developmental anomalies of the tracheal mesenchyme, especially the cartilage and SM, result in severe airway disorders, including tracheomalacia and tracheostenosis. Over the past three decades, various animal models have been generated to mimic congenital malformations of the human trachea (Table 1) and key genes have been identified that are associated with the etiology of such airway defects, including HH, WNT and FGF signaling components.

**Tracheomalacia** is the most common congenital tracheal deformity with an incidence of 1 in 2100 children (Boogaard et al., 2005). In tracheomalacia, the trachea is softened and floppy because of the abnormal cartilage development. Tracheomalacia can occur alone; however, it is frequently associated with various congenital disorders, such as Pallister–Hall syndrome, Pfeiffer syndrome and tracheoesophageal fistula (Carden et al., 2005). Various germline mutations to HH-responsive genes and FGF signaling have been identified (e.g. *GLI3* in Pallister–Hall syndrome, and *FGFR1* and *FGFR2* in Pfeiffer syndrome; Nasr et al., 2021).

**Tracheostenosis** is a rare congenital disorder with an incidence of approximately 1 in 64,500 infants. It is characterized by abnormal narrowing of the trachea, resulting in breathing difficulties. The most common feature of tracheostenosis is complete or near-complete tracheal ring deformity (CTRD), in which the tracheal cartilage extends to dorsal aspects, thereby preventing normal tracheal growth. In many cases, tracheostenosis is associated with Pfeiffer syndrome and cardiovascular diseases involving the pulmonary artery. Whole-exome sequencing of patients with CTRD and their parents identified several causative genes associated with HH signaling (i.e. *SHH*, *HSPG2*) and WNT signaling (i.e. *ROR2*) (Sinner et al., 2019).

**A tracheal cartilaginous sleeve**, caused by the fusion of crescent-shaped cartilage and loss of fibroblasts between cartilaginous rings that results in a single continuous cartilage sheet, is associated with craniosynostoses, including Pfeiffer syndrome, Apert syndrome and Crouzon syndrome, and are caused by mutations in FGF signaling genes (i.e. *FGFR1*, *FGFR2*, *FGF10*) (Lertsburapa et al., 2010).

**Table 1.**
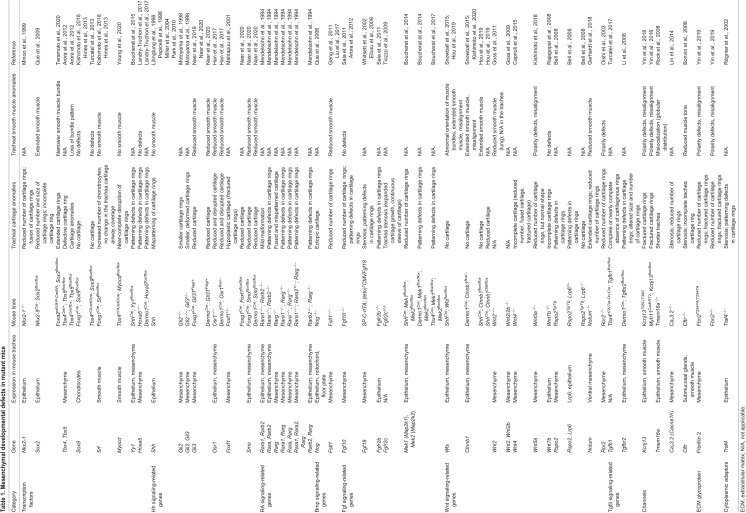
Mesenchymal developmental defects in mutant mice

## Tracheal development

**Fig. 2.**
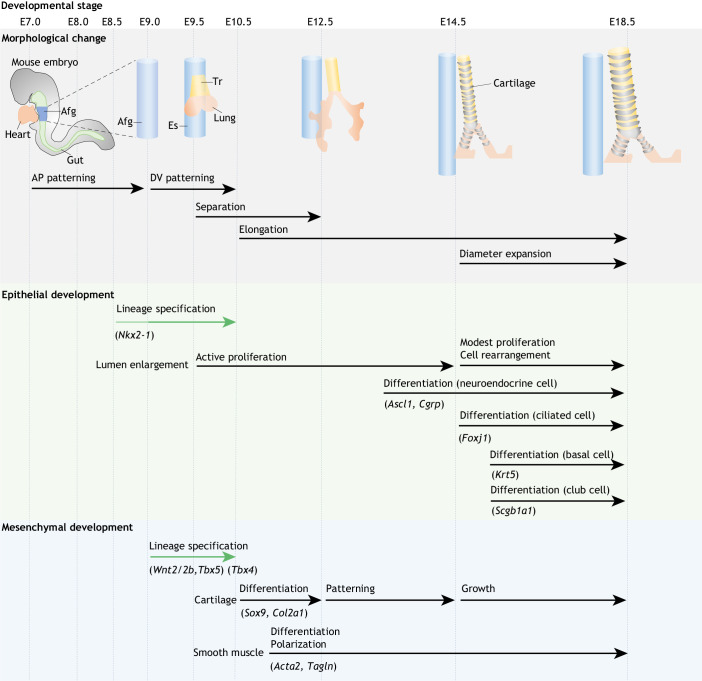
**An overview of mouse trachea development.** Tracheal development progresses in sequential events of AP patterning, DV patterning, separation and tube elongation/diameter expansion. The initiation of lineage specification in the epithelium takes place around E8.5-9.0. Serls and colleagues described the earliest expression of *Nkx2-1*, but the majority of the studies demonstrated that *Nkx2-1* expression is first detected around E9.0 (Cardoso and Lü, 2006; Lazzaro et al., 1991; Nikolić et al., 2018; Serls et al., 2005). In all processes, there is a high level of communication between the epithelium and surrounding mesenchyme to promote accurate development of the tubular structures. Afg, anterior foregut; AP, anterior-posterior; DV, dorsal-ventral; Es, esophagus; Tr, trachea.

The development of the mouse trachea is divided into four sequential processes: regionalization with anterior-posterior (AP) patterning [embryonic day (E) 7.0-8.5], lineage specification with dorsal-ventral (DV) patterning (E8.5-10.5), tube separation (E9.5-12.5) and tube elongation/expansion (E10.5-18.5) (Kishimoto et al., 2018; Nasr et al., 2019; Serls et al., 2005; Zorn and Wells, 2009) (Fig. 2). The stages of AP and DV patterning are common in both tracheal and lung formation, whereas tube separation and elongation/expansion are unique to the tracheal development.

Briefly, tracheal development in the mouse begins following the regionalization of the primitive gut tube to the foregut at E7.0-8.5. Subsequently, the anterior foregut endoderm (AFE) is patterned along the DV axis by the ventral expression of NK2 homeobox 1 (*Nkx2-1*) and the dorsal expression of SRY-box transcription factor 2 (*Sox2*) at ∼E9.0 (Kaufman, 1992; Que, 2015; Serls et al., 2005). *Nkx2-1* is among the earliest and most reliable markers of the tracheal/lung epithelial progenitors and the thyroid. Nkx2-1^+^ cells appear in the ventral AFE (vAFE) and promote the development of the primary lung buds at the bilateral side of the vAFE (Elluru et al., 2009; Minoo et al., 1999). Just after the lung buds emerge, the primordial trachea appears at the vAFE above the lung buds. Following lineage specification, the trachea undergoes stepwise morphogenesis processes of tube separation, elongation and diameter expansion. The tracheoesophageal separation process, which occurs between E9.5 and E12.5 (Billmyre et al., 2015; Que, 2015), is orchestrated by mutual endoderm-mesoderm interactions with retinoic acid (RA), sonic hedgehog (Shh), wingless-type MMTV integration site family member 2/2b (Wnt2/2b) and bone morphogenetic protein 4 (Bmp4) (Morrisey and Hogan, 2010; Rankin and Zorn, 2014). During the separation process, the trachea begins to elongate along the AP axis with no widening of the diameter, which is followed by diameter expansion in parallel with cartilage growth (Kishimoto et al., 2018) (Fig. 2). Tracheal morphology is determined during the elongation and expansion stages, which are initiated by the mesenchymal tissues, including the cartilage and SM.

### Regionalization with AP patterning

As a prologue to the development of the respiratory organs, the regional identity of the endoderm along the AP axis is specified by interactions between the primitive gut endoderm and surrounding mesoderm from E7.0. The process of AP patterning has been extensively analyzed in various animal models, including mouse, frog and zebrafish. During mouse development, mesoderm-derived Wnt, Bmp and fibroblast growth factor (Fgf) induce posterior identity via the activation of caudal-type homeobox (*Cdx*) in the posterior endoderm, while suppressing anterior endoderm expression of hematopoietically expressed homeobox (*Hhex*) and *Sox2* (Zorn and Wells, 2009). The position of the tracheal primordium is reportedly influenced by the adjacent cardiac tissue (Serls et al., 2005). *Ex vivo* culture experiments showed that the foregut endoderm can differentiate into Nkx2-1^+^ respiratory epithelial progenitors in the presence of cardiac tissue.

Cardiac-derived Fgf2 could be a cue for *Nkx2-1* induction, because recombinant Fgf2 was found to efficiently induce *Nkx2-1* expression in mouse foregut culture. However, during *in vitro* differentiation of human pluripotent stem cells (PSCs), the fate of the thyroid, rather than that of the respiratory tract, is promoted in the presence of Fgf2 (Dye et al., 2015; Kurmann et al., 2015; Serra et al., 2017). Additionally, the specification of the tracheal epithelium was not inhibited in other knockout mice of the Fgf family (e.g. *Fgf10* and *Fgfr2*), as well as in cultured embryos treated with Fgf blockers (Hou et al., 2019; Hyatt et al., 2004; Sekine et al., 1999). These results imply that the role of Fgf signaling in *Nkx2-1* induction remains controversial.

**Fig. 3.**
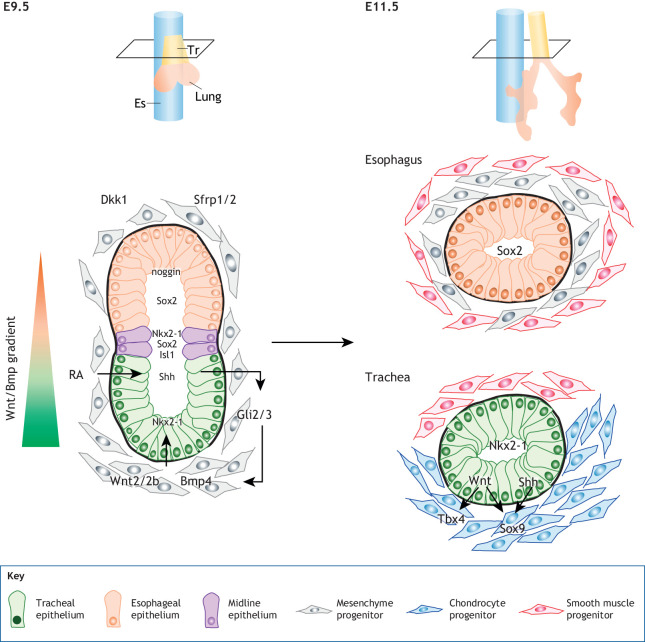
**DV patterning of the AFE.** A series of mutual interactions between the endoderm and mesoderm initiates *Nkx2-1* expression in the ventral anterior endoderm whereas the dorsal endoderm expresses *Sox2*. Retinoic acid (RA), sonic hedgehog (Shh), Wnt2/2b and bone morphogenetic protein 4 (Bmp4) play central roles in DV patterning. A few Nkx2-1^+^/Sox2^+^/Isl1^+^ cell populations are present at the boundary of these DV domains. After separation, Wnt ligands in the endoderm induce mesenchymal *Tbx4* expression to ensure tracheal mesenchyme identity. Chondrogenic *Sox9* expression is induced by endodermal Shh and Wnts. Es, esophagus; Tr, trachea.

As *Nkx2-1* has been identified as the earliest marker and necessary for respiratory epithelial progenitors, one of the most fascinating topics in the field of developmental biology is the regulatory gene networks that initiate *Nkx2-1* expression (Lazzaro et al., 1991; Minoo et al., 1999). *Nkx2-1* expression is orchestrated by endoderm-mesoderm interactions modulated by RA, Hh, Wnt and Bmp signaling (Fig. 3). During AP patterning, the RA synthesis enzymes retinaldehyde dehydrogenase 2 and retinol dehydrogenase 10 are enriched in putative respiratory fields of lateral plate mesoderm. Loss-of-function experiments for RA production using *Xenopus* and mouse embryos revealed that RA patterns the lateral plate mesoderm to presumptive lung mesoderm, which expresses *Wnt2/2b* (Rankin et al., 2016). Then, RA triggers endodermal *Shh* expression, which induces *Wnt2/2b* and *Bmp4* in mesoderm and, subsequently, *Nkx2-1* in epithelium. Thus, RA production in the anterior foregut is a prerequisite for *Nkx2-1* induction (Litingtung et al., 1998; Molotkov et al., 2005; Motoyama et al., 1998; Nasr et al., 2019; Rankin et al., 2016).

### Lineage specification with DV patterning

After AP patterning, tracheal/lung progenitors emerge at the vAFE according to the DV pattern. During DV patterning from E8.5 to E10.5, the naïve foregut endoderm is compartmentalized into the ventral Nkx2-1^+^ domain and dorsal Sox2^+^ domain (Lazzaro et al., 1991; Minoo et al., 1999; Serls et al., 2005) (Fig. 3). Analyses of *Sox2* mutants demonstrated that Sox2 regulates DV patterning in a dose-dependent manner (Que et al., 2007; Trisno et al., 2018). In contrast, the *Nkx2-1* mutant ventrally expands the Sox2^+^ domain, resulting in defective DV patterning. These findings indicate that the mutual repression between Nkx2-1 and Sox2 coordinates proper DV patterning. At the boundary of these DV domains, there are a few Nkx2-1^+^/Sox2^+^/ISL LIM homeobox 1 (Isl1)^+^ cell populations that are indispensable for tracheoesophageal separation, because endodermal deletion of Isl1 results in tracheoesophageal separation defects with a decrease in the midline population of Nkx2-1^+^/Sox2^+^ cells (Han et al., 2020; Kim et al., 2019; Nasr et al., 2019).

Lineage specification with DV patterning is accompanied by the establishment of a Wnt/Bmp gradient along the DV axis (Fig. 3). At this stage, *Wnt2/2b* is expressed in the ventral-lateral mesoderm. Genetic ablation of *Wnt2/2b* results in complete tracheal agenesis and loss of *Nkx2-1* expression. Supporting this observation, endoderm-specific deletion of *Ctnnb1*, a core component of the canonical Wnt signaling pathway, also results in complete loss of *Nkx2-1* expression in the respiratory system, although *Nkx2-1* expression in the thyroid is unaffected. Thus, mesodermal Wnt2/2b ligands activate canonical Wnt signaling in the endoderm to induce *Nkx2-1* expression in the airway (Goss et al., 2009; Harris-Johnson et al., 2009). Recently, Gerner-Mauro and colleagues investigated whether a quadruple Tcf/Lef paralog (*Tcf7/Tcf7l1/Tcf7l2/Lef1*) had a redundant and/or additive role in the development of respiratory organs (Gerner-Mauro et al., 2020). Endoderm-specific knockout of the quadruple Tcf/Lef paralog led to a tracheoesophageal fistula phenotype, demonstrating that the Wnt2/2b-β-catenin-Tcf/Lef paralog axis is required for DV patterning. In contrast, Wnt signaling is downregulated in the dorsal anterior foregut, which expresses several Wnt antagonists, including dickkopf WNT signaling pathway inhibitor 1 (*Dkk1*) and secreted frizzled related protein 1/2 (*Sfrp1/2*). It has also been reported that BARX homeobox 1 (Barx1) negatively regulates *Sfrp1/2* in the dorsal mesenchyme, and its deletion leads to excessive Wnt activation and tracheoesophageal fistula (Woo et al., 2011). Additionally, endodermal *Sox2* expression has been shown to increase the expression of Wnt antagonists in the dorsal anterior foregut (Trisno et al., 2018). Although the exact mechanism remains unclear, Sox2 might utilize Barx1 to regulate the expression of Wnt antagonists.

The Bmp signaling pathway is also crucial in DV patterning (Fig. 3). *Bmp4* is expressed in the vAFE and the surrounding splanchnic mesoderm. *Bmp4* knockout resulted in tracheal agenesis in mice but had no effect on tracheal lineage specification (Li et al., 2008). In contrast, double knockout of *Bmpr1a:b* resulted in tracheal agenesis and reduced *Nkx2-1* expression (Domyan et al., 2011), suggesting that other Bmp ligands might play a redundant role in *Nkx2-1* expression. Bmp signaling is downregulated in the dorsal foregut because of the expression of noggin (a Bmp antagonist) in the dorsal endoderm, notochord and floor plate (Que et al., 2006). In mice, the lack of noggin leads to esophageal agenesis (Que et al., 2006). Analyses of tissue-specific knockout mice revealed that the lack of notochord-derived noggin results in esophageal atresia with tracheoesophageal fistula following the defect of DV patterning (Fausett et al., 2014; Que et al., 2006). The combinatorial knockout of *Nog* and *Bmp4* or *Bmp7* (*Nog*^−/−^, *Bmp4*^+/−^ or *Nog*^−/−^, *Bmp7*^−/−^) rescues the phenotype of tracheoesophageal fistula, demonstrating the importance of the balancing of Bmp signaling by agonists and antagonist (Li et al., 2008; Que et al., 2006). Bmp signaling most likely suppresses *Sox2* expression to establish the DV pattern, as the disruption of Bmp signaling increases *Sox2* expression in the ventral endoderm (Domyan et al., 2011).

Current studies have revealed that Nkx2-1 is not an exclusive factor driving tracheal/lung development. Kuwahara and colleagues revealed that many respiratory epithelium progenitor genes are not controlled by Nkx2-1 (Kuwahara et al., 2020). Furthermore, we reported that the expression of T-box transcription factor 4 (*Tbx4*), a mesenchymal marker of the trachea and lung, is retained in the ventral mesoderm of *Nkx2-1*-null mice (Kishimoto et al., 2020). In parallel with *Nkx2-1* induction, bidirectional Wnt signaling between the vAFE and foregut mesoderm plays an important role independent of Nkx2-1 (Kishimoto et al., 2018).

### Models of tracheoesophageal tube separation

Tracheoesophageal separation is a dynamic morphogenetic process that is coordinated by multiple cellular events of differentiation, proliferation, condensation and rearrangement. In a recent study, the sequential cellular events that are responsible for tracheoesophageal separation were clearly defined in mouse and frog models (Nasr et al., 2019). Tracheoesophageal separation involves five events: DV patterning, medial constriction, epithelial fusion and remodeling, breakdown of the extracellular matrix and mesenchymal invasion.

**Fig. 4.**
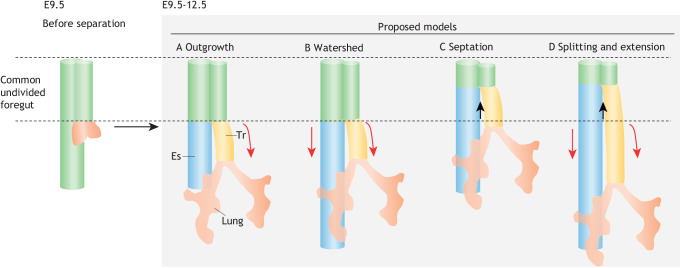
**Models of tracheoesophageal separation.** Four models of tracheoesophageal separation are currently proposed. (A) In the outgrowth model, the trachea forms from the common foregut and then elongates (red arrows). (B) In the watershed model, the trachea and esophagus individually elongate. However, the common undivided foregut does not split in these models. (C) In the septation model, a septum emerges at the DV boundary and superiorly moves to separate the common undivided foregut (black arrows). (D) In the splitting/extension model, the nascent trachea and esophagus elongate and the common undivided foregut is separated. Es, esophagus; Tr, trachea.

Although there is currently no consensus on the most appropriate model of tracheoesophageal separation at the anterior foregut, four models have been proposed: an outgrowth model, in which tracheal/lung progenitors form a protruding diverticum to generate a primordial tracheal tube at the vAFE, followed by proximal to distal elongation of the tracheal tube, while the remaining common dorsal anterior foregut forms the esophagus (Fig. 4A); a watershed model, in which both the trachea and esophagus are elongated with no change in the length of the common undivided foregut (Fig. 4B); a septation model, whereby septa appear at both the lateral side of the DV midline region of the anterior foregut and vertically grow inside and fuse at the tips to form one large septum, which rostrally moves and divides the common undivided foregut into the trachea and esophagus, resulting in decreased length of the common undivided foregut and increased lengths of the trachea and esophagus (Fig. 4C); and a splitting/extension model, whereby the DV midline divides the common undivided foregut into the nascent trachea and esophagus, which caudally elongate individually (Fig. 4D) (Billmyre et al., 2015; Ioannides et al., 2010; Que, 2015).

To determine the most accurate model, quantifying changes to the lengths of the trachea, esophagus and common undivided foregut is important. The common undivided foregut shortens between E10.5 and E12.5 whereas the separated trachea elongates, which refutes the events in the outgrowth and watershed models (Ioannides et al., 2010). It has been reported that the length of the common undivided foregut decreases with simultaneous increases with both the trachea and esophagus (Que, 2015; Nasr et al., 2019). Based on these findings, the splitting/extension model (Fig. 4D) seems to be the most accurate.

### Mesenchymal tissues regulate tube elongation and expansion

#### Regulated mesenchymal tissue differentiation shapes the respiratory and gastrointestinal tracts

We have recently identified that the length and diameter of the murine trachea are regulated by distinct mechanisms (Kishimoto et al., 2018). Quantitative morphological analysis of tracheal tubulogenesis revealed that the developing trachea elongates its length with no change in diameter between E10.5 and E14.5 (Fig. 2). Interestingly, genetic ablation of *Wnt5a* and receptor tyrosine kinase-like orphan receptor 2 (*Ror2*) shortened tracheal length with little change in diameter. Wnt5a-Ror2 signaling is required for synchronized mesenchymal cell polarity in SM progenitors, named ‘radial cell polarity’, which restricts diameter expansion and simultaneously promotes tube elongation (Kishimoto et al., 2018). Yin and colleagues reported that defective mesenchymal polarization by mutations to fibrillin 2 (*Fbn2*) or potassium inwardly rectifying channel subfamily J member 13 (*Kcnj13*) causes a short trachea phenotype (Yin et al., 2019, 2018). Furthermore, the loss of Tmem16a (*Ano1*) leads to disorganization of the SM tissue structure, resulting in a shorter tracheal phenotype (Rock et al., 2008). Thus, radial polarization of SM progenitors is required for tube elongation. Of note, recent studies reported that mutant mice with undifferentiated tracheal SM cells do not exhibit the shorter trachea phenotype, suggesting that polarization, rather than differentiation, is required for tube elongation (Kishimoto et al., 2018; Young et al., 2020). From E14.5, the diameter of the trachea expands along with continuous tube elongation coincident with cartilage growth. Human developmental anomalies of the tracheal cartilages lead to obstructive defects, such as tracheostenosis and tracheomalacia (Box 2). In animal models, several groups have reported that disruption of *Sox9* results in the absence of tracheal chondrocytes and subsequent failure of diameter expansion (Kishimoto et al., 2018; Turcatel et al., 2013). In parallel with these insights, a previous report demonstrated that overexpression of *Fgf18* leads to tracheal cartilage mispatterning, resulting in a narrow trachea (Elluru et al., 2009). To summarize, the structure and stiffness of the cartilage promote diameter expansion.

#### Luminal epithelial morphogenesis

During the tube elongation and expansion stages, the epithelial surface expands and develops into pseudostratified epithelial layers at the lumen. In developmental biology, the cellular basis determining the lumen size of the major organs of the digestive and respiratory systems has not yet been elucidated. The lumen size of the developing mouse trachea is temporally controlled by the combination of cell proliferation, changes in the cell shape, and cell rearrangement (Kishimoto et al., 2018). Until E14.5, the increase in lumen size correlates well with that of cell numbers, suggesting that cell proliferation is the major driving force that underlies lumen enlargement. After E14.5, cell proliferation decreases, whereas luminal size increases by apical emergence, expansion of the apical surface and modest cell proliferation (Kishimoto et al., 2018). Importantly, such epithelial rearrangement is controlled by the surrounding cartilaginous rings. Either disrupted differentiation or reduced stiffness of the cartilage inhibits epithelial rearrangement (Kishimoto et al., 2018).

Lumen morphogenesis of the trachea and lungs are quite different. In terms of spatial regulation of cell proliferation, there is no particular location of proliferation in the developing tracheal epithelium, whereas mitotic epithelial cells are restricted at the tips of the lung buds (Kishimoto et al., 2018; Okubo et al., 2005). As described above, the proliferation of epithelial cells is synchronously decreased after E14.5, suggesting that the proliferation capacity is temporally, but not spatially, regulated in the developing trachea. Although both the trachea and the lung airways elongate along the AP axis at early stages (Kishimoto et al., 2018; Tang et al., 2011, 2018), the regulating mechanisms are not the same. During early development, the epithelial morphogenesis of the trachea and lungs mainly relies on cell proliferation. Indeed, in the lungs, oriented cell division allows the lumen to grow along the AP axis (Tang et al., 2011). In contrast, the epithelial cells of the trachea exhibit random orientations. Dynamic changes in cell shape and alignment might enable elongation along the AP axis in the trachea (Kishimoto et al., 2018).

### The epithelial cell differentiation

In parallel with luminal enlargement, naïve epithelial progenitor cells differentiate into specialized cell types, coinciding with cell cycle slow-down after E13.5 (Kiyokawa et al., 2021) (Fig. 2). Neuroendocrine cells are the earliest cell types expressing lineage-specific transcriptional factors, such as *Ascl1* and *Cgrp*, from E13.5 (Kiyokawa et al., 2021). Subsequently, Foxj1^+^ ciliated cells appear around E14.5 whereas Krt5^+^ basal cells and Scgb1a1^+^ club cells can be observed from E15.5 (Kiyokawa et al., 2021; Rawlins et al., 2007). The differentiation of these epithelial cells is coordinated by various signaling pathways, including Notch, Fgf, Tgfβ and Bmp signaling (Hou et al., 2019; Kiyokawa et al., 2021; Morimoto et al., 2010, 2012; Mou et al., 2016; Tsao et al., 2009; Turcatel et al., 2017). Importantly, manipulation of these signaling pathways is a common approach to direct differentiation from PSCs to the cell types of interest. The detailed mechanisms of epithelial differentiation have been reviewed by Swarr and Morrisey (2015).

### Role of mechanical forces

Mechanical characteristics of the cartilage and SM tissues contribute to tracheal development. For example, mesenchymal progenitors might exert force that contributes to elongation of the tracheal tube without expansion until E14.5 as the radial cell polarity of the SM progenitor cells coordinates the contractile force inward that reduces tube diameter (Kishimoto et al., 2018). In addition, two independent studies suggested that disruption of SM development leads to cartilage defects, probably induced by disrupted tensile elasticity (Yin et al., 2018; Young et al., 2020). Hence, SM progenitors provide force to regulate tube shape and cartilage tissue. After E14.5, cartilage stiffness contributes to diameter expansion, as described above (Kishimoto et al., 2018). Therefore, mesenchymal populations play pivotal roles in trachea tissue morphogenesis via the mechanical character of the cartilage and SM.

### Mechanisms underlying mesenchymal development

Although the mesenchyme is a key feature of organogenesis, epithelial development continues to be the main focus in the field of organogenesis. In the trachea, normal mesenchymal development, especially of the cartilage and SM, is crucial for shape determination and epithelial rearrangement (Fig. 2). Here, the signaling pathways that underlie the development of the tracheal mesenchyme are summarized.

#### Single-cell RNA transcriptome of the developing mesenchyme

Recent investigations have determined the cell fates and differentiation processes of fetal tracheal/lung mesenchymal cells. Single-cell transcriptome analyses have facilitated comprehensive investigations of developing mesenchymal cells. Han and colleagues successfully delineated a developmental roadmap of the visceral mesoderm from E8.5 to E9.5, which deciphered the lineages of organ-specific mesenchymal cells (Han et al., 2020). Specification of the tracheal/lung mesenchyme occurs within the lateral plate mesoderm from E9.0 to E9.5. Hence, tracheal/lung mesenchymal progenitors can be defined by combinational expression of the *Nkx6-1/Gata4/Wnt2* genes, but not by a single marker gene, such as *Nkx2-1*, in the respiratory endoderm. Furthermore, *in silico* metagene expression profile analysis predicted that RA/Bmp/Hh signaling is activated in the tracheal/lung mesenchyme lineage, which is consistent with previous mouse genetics studies (Table 1). By contrast, Wnt2^+^/GLI family zinc finger 1 (Gli1)^+^/Isl1^+^ cardiopulmonary progenitors are reported to give rise to the lung mesenchyme, including the vascular and airway SM and pericyte-like cells (Peng et al., 2013). However, the contribution of this population to tracheal mesenchyme lineages remains unclear.

#### Specification of Tbx4/5^+^ tracheal mesenchyme

Tbx4 and Tbx5 are established genetic markers of the respiratory mesenchyme and play central developmental roles (Arora et al., 2012; Steimle et al., 2018). *Tbx5* expression is observed earlier than E9.0, whereas Tbx4 appears during tracheoesophageal separation around E10.5, implying that *Tbx4* expression ensures tracheal mesenchyme identity after E10.5. Canonical Wnt signaling was identified as the upstream signal of *Tbx4* expression (Kishimoto et al., 2020). Mesenchymal *Ctnnb1* deletion disrupts Tbx4 expression, resulting in trachea agenesis by E16.5. Interestingly, endodermal deletion of the Wnt ligand secretion mediator gene (*Wls*), which encodes a cargo receptor required for Wnt ligand secretion, also causes loss of Tbx4, suggesting that an endodermal Wnt ligand induces *Tbx4* expression in the mesoderm (Kishimoto et al., 2020). In addition to the initial specification of mesenchyme, Wls is further required at later stages of mesenchymal development, including chondrogenesis and SM orientation (Snowball et al., 2015). Considering that mesenchymal Wnt2/2b initiates specification of the tracheal endoderm, bidirectional Wnt signaling between the endoderm and mesoderm enables the lateral plate mesoderm to acquire the tracheal mesenchyme phenotype. In the lung, unlike the trachea, Tbx4 is not under the control of bidirectional Wnt signaling because *Tbx4* expression was retained after the deletion of mesenchyme-specific *Ctnnb1*, suggesting that the machinery governing specification of the tracheal mesenchyme is distinct from that of the lung.

### Tracheal cartilage development

**Fig. 5.**
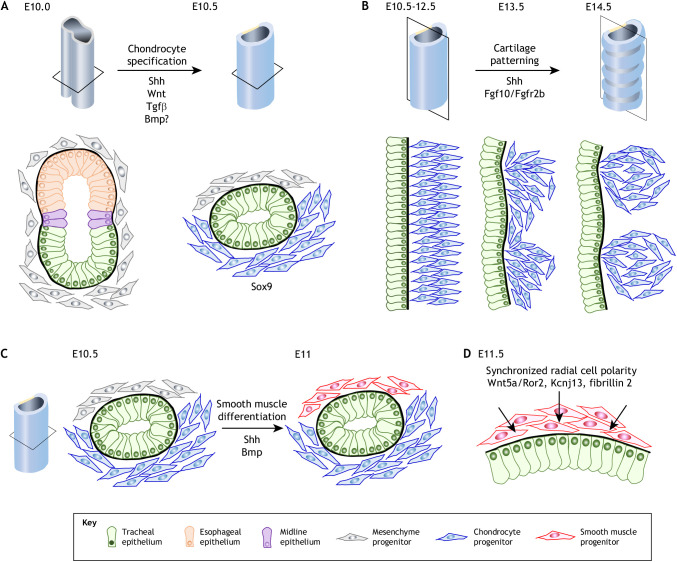
**Differentiation and patterning of mesenchyme.** (A) Transverse view of the trachea showing the differentiation of tracheal chondrocytes. Ventral-lateral mesenchyme progenitors differentiate into Sox9^+^ chondrocyte progenitors. Sonic hedgehog (Shh), Wnt and transforming growth factor beta 1 (Tgfβ1) signaling promotes *Sox9* expression to initiate differentiation. (B) Sagittal view of the trachea showing the distribution of Sox9^+^ chondrocytes. The progenitors periodically condensate and segregate along the AP axis from E12.5 to E14.5. Fibroblast growth factor (Fgf) and Hedgehog (Hh) signaling is involved in the patterning. (C) Transverse view of the trachea showing the differentiation of the tracheal smooth muscle (SM). Dorsal mesenchyme progenitors differentiate into SM progenitors. The activation of Shh and inhibition of bone morphogenetic protein (Bmp) signaling induce differentiation of the tracheal SM. (D) Transverse view of the trachea showing synchronized radial polarization of tracheal SM progenitors, which requires Wnt5a/Ror2, Kcnj13 and fibrillin 2.

The human and mouse trachea contain 16-22 and 13-15 cartilaginous rings, respectively (see Fig. 1A), which prevent collapse of the airway. Recent studies showed that cartilage formation is required for normal tracheal development, including epithelial cell differentiation, rearrangement and diameter expansion (Hines et al., 2013; Hou et al., 2019; Kishimoto et al., 2018). Lineage-tracing experiments using forkhead box G1 (*Foxg1*)^Cre^ and *Wnt1^Cre^* drivers showed that chondrocyte progenitors arise from the lateral plate mesoderm, but not the neural crest population (Nasr et al., 2021). Genetic markers of chondrocytes, such as *Sox9*, appear in the ventral-lateral plate mesoderm just after tracheoesophageal segregation in the mouse at E10.5 (Nasr et al., 2021) (Fig. 5A). Sox9 is a high-mobility group domain transcriptional factor crucial for chondrogenesis and directly upregulates chondrogenic genes, such as collagen type II alpha 1 chain and aggrecan (Bell et al., 1997; Bi et al., 1999; Sekiya et al., 2000). Therefore, mice lacking *Sox9* exhibit the trachea cartilage agenesis phenotype (Hines et al., 2013; Kishimoto et al., 2018; Turcatel et al., 2013).

Several key molecules that participate in the regulation of tracheal chondrogenesis have been identified by mouse genetic studies (Table 1). The phenotypes of these mutant mice indicate that Wnt, Hh and transforming growth factor beta 1 (Tgfβ1) signaling regulate *Sox9* expression and chondrocyte specification. Mesodermal knockout of *Ctnnb1* results in loss of Sox9^+^ chondrocyte progenitors. This phenotype is most likely caused by disrupted identity of the tracheal mesenchyme due to the absence of Tbx4 (Hou et al., 2019; Kishimoto et al., 2020; Snowball et al., 2015). Disruption of Hh signaling components also alters the development of Sox9^+^ chondrocyte progenitors (Miller et al., 2004; Motoyama et al., 1998; Park et al., 2010; Pepicelli et al., 1998). Hh signaling is required for chondrocyte proliferation, differentiation and patterning. Nasr and colleagues recently reported that Hh signaling regulates expression of Wnt signaling components, suggesting that cross-talk between Wnt and Hh coordinates chondrocyte development (Nasr et al., 2021). In addition, Tgfβ1 seems to be involved in chondrocyte development because mesenchymal deletion of *Tgfb1* dramatically reduces *Sox9* expression (Turcatel et al., 2017), yet the mechanism that underlies the integration of Tgfβ1 signaling and *Sox9* expression is unclear. In general, Bmp signaling can induce *Sox9* expression in the developing chondrocyte (Yoon and Lyons, 2004). Nevertheless, the role of Bmp signaling in tracheal chondrogenesis remains unclear because a deficiency in Bmp signaling results in tracheal agenesis. Hence, a spatiotemporally regulated conditional knockout strategy should be utilized to analyze the role of Bmp signaling on tracheal chondrogenesis.

Sox9^+^ progenitors align in the subepithelium of the ventral-lateral mesenchyme at E10.5-12.5. Around E13.5, these cells create several crescent-shaped condensates exhibiting periodic patterns along the AP axis (Fig. 5A,B) (Sala et al., 2011; Yoshida et al., 2020). Although the detailed mechanisms that underlie this condensation and patterning have not been fully elucidated, several genes and signaling networks, including Hh and Fgf, seem to orchestrate these processes (Table 1). Mesoderm-derived Fgf10 binds endodermal Fgfr2 to control the amplitude of periodicity of endodermal *Shh* expression around E13.5 (Sala et al., 2011). Therefore, *Fgf10* and *Shh* under- or overexpression results in disrupted patterning of the cartilaginous rings. This is consistent with genetic evidence that several Fgf mutants have a cartilaginous tracheal sleeve, which is a rare congenital airway malformation (Table 1). Furthermore, mice lacking the Hh-target genes *Foxf1*, odd-skipped related transcription factor 1 (*Osr1*) and *Gli1* also exhibit altered cartilage structures.

#### Tracheal SM development

In the trachea, SM located on the dorsal aspect of the mesenchyme connects the ends of cartilaginous rings. However, the role of SM during normal development remains unclear. Interestingly, Young and colleagues reported that mice lacking SM differentiation failed to generate mature cartilage. Normally developing Sox9^+^ chondrocyte progenitors are present in the mutant at E13.5, but the proportion dramatically decreases after E14.5 (Young et al., 2020). Moreover, the neural tissues and vasculature of the mutants are disorganized. A similar cartilage defect was also observed in mice with a mutation in myosin light chain kinase, suggesting that the mechanical properties of SM maintain and promote cartilage growth.

The development of the tracheal SM begins around E11 with the expression of marker genes, such as actin alpha 2 (*Acta2*) and transgelin (Tollet et al., 2001) under the regulation of the key transcriptional factors serum response factor (Srf) and myocardin (Hines et al., 2013; Young et al., 2020) (Fig. 5C). Hh and Bmp are common signaling molecules that regulate SM differentiation in various organs. Genetic ablation of *Shh* and its target genes *Foxf1* and *Osr1* leads to obvious reductions in airway SM in both the trachea and lung (Han et al., 2017; Miller et al., 2004; Pepicelli et al., 1998; Ramalho-Santos et al., 2000; Ustiyan et al., 2018). In the intestine, the Hh gradient determines SM patterning by inhibiting Bmp signaling (Huycke et al., 2019). Although the effect of Bmp ligands on tracheal SM differentiation is unclear, loss of follistatin-like 1 (a Bmp antagonist) reduced tracheal SM (Liu et al., 2017). Similar to intestinal development, Hh might inhibit Bmp signaling in the trachea to induce SM differentiation. Interestingly, contrary to the conserved role of Shh and Bmp signaling in SM development in other organs, the role of Wnt and Fgf signaling seems to differ among organs. For example, Wnt2 positively regulates SM differentiation via the activation of *Fgf10* and *Wnt7b* expression in the lung, but disruption of Wnt signaling ventrally expands tracheal SM (Kishimoto et al., 2020). Moreover, tracheal SM is normally differentiated and aligned in *Fgf10* knockout mice (Sala et al., 2011). Taken together, tracheal SM differentiation requires Hh activation and Bmp inhibition but not Wnt and Fgf signaling.

Upon differentiation, the cell polarity of SM progenitors is synchronized and directed toward the tubular epithelium in a process referred to as radial cell polarity. Synchronized polarity is needed to align SM bundles below the epithelial tissue (Kishimoto et al., 2018) (Fig. 5D). However, in Srf-null mice, complete loss of SM differentiation had no effect on synchronized radial cell polarity, indicating that polarity regulation is independent of differentiation. Radial polarization of SM progenitors is regulated by the Wnt5a/Ror2 axis, and genetic ablation of *Wnt5a* or *Ror2* leads to random polarization of SM cells and a shorter trachea, demonstrating that cell polarity, but not differentiation, is crucial for tube elongation. Similarly, the loss of *Tbx4/5*, *Kcnj13*, *Cftr*, *Tmem16a* and fibrillin 2 causes misalignment of SM bundles (Table 1) (Arora et al., 2012; Bonvin et al., 2008; Kishimoto et al., 2018; Rock et al., 2008; Yin et al., 2019, 2018). Although SM cells radially polarize toward epithelial cells, these genes are expressed in SM, but not the epithelium, suggesting permissive roles of these genes on polarization. It is possible that other instructive cues are secreted from epithelial tissue. Thus, future studies are warranted to identify potential instructive epithelial factors.

## *In vitro* reconstruction of respiratory tissue

**Fig. 6.**
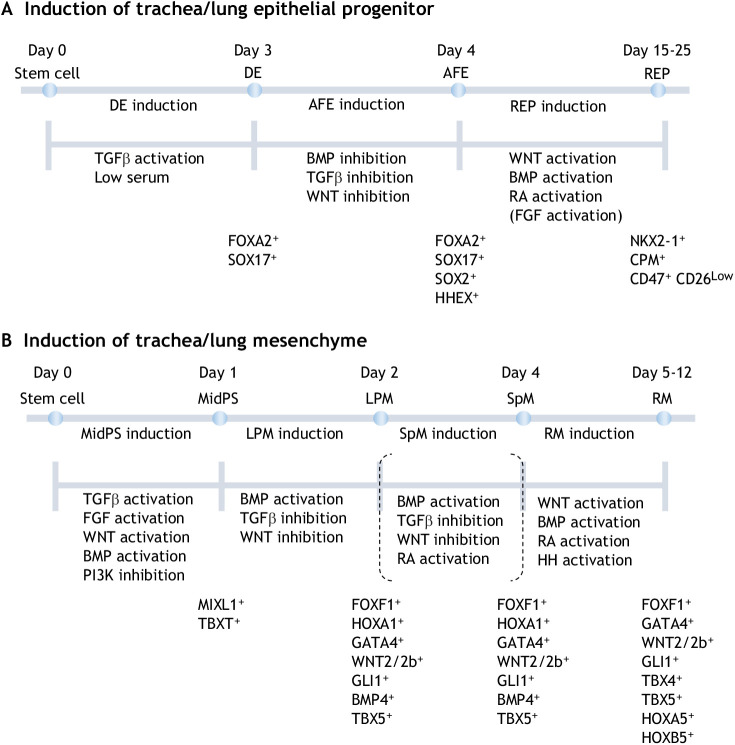
***In vitro* reconstruction of the tracheal/lung epithelium and mesenchyme.** (A) Directed differentiation to respiratory epithelial progenitors. Embryonic stem cells (ESCs) and induced pluripotent stem cells (iPSCs) differentiate into FOXA2^+^/SOX17^+^ definitive endoderm (DE) and then are converted into FOXA2^+^/SOX2^+^ anterior foregut endoderm (AFE). Subsequently, NKX2-1^+^ respiratory epithelial progenitors (REP) are induced via the activation of the retinoic acid (RA), WNT and bone morphogenetic protein (BMP) pathway. (B) Directed differentiation to respiratory mesenchyme. ESCs and iPSCs differentiate into the middle primitive streak (MidPS) and are then converted into the FOXF1^+^/VIM^+^ lateral plate mesoderm (LPM), which is further regionalized into the splanchnic mesoderm (SpM) with RA activation. The tracheal/lung mesenchyme is induced via the activation of the RA, Hedgehog (HH) and BMP pathway. RM, respiratory mesenchyme. CD26 (DPP4); T (TBXT).

The reconstruction of respiratory organs from human PSCs is a tremendous tool for studying human developmental biology. An organoid grown from PSCs in the lab was recently reconstructed by experimentally mimicking the process of *in vivo* organogenesis in a culture dish (McCracken et al., 2014; Múnera et al., 2017; Spence et al., 2011; Trisno et al., 2018). For *in vitro* reconstruction of tracheal/lung epithelial cells, embryonic stem cells (ESCs) and induced pluripotent stem cells (iPSCs) were sequentially differentiated into primitive streak, definitive endoderm, AFE and tracheal/lung epithelial cells (Fig. 6A). For mesenchymal differentiation, similar to this concept, ESCs or iPSCs are converted into middle primitive streak cells, then lateral plate mesoderm and, finally, tracheal/lung mesenchyme (Han et al., 2020) (Fig. 6B).

### Reconstruction of respiratory epithelium

#### PSCs to definitive endoderm differentiation

In the early embryo, nodal, a member of the Tgfβ superfamily, plays a central role in endoderm specification through SMAD (Lowe et al., 2001; Zorn and Wells, 2009). WNT, BMP and FGF also modulate nodal-induced endoderm specification (Tam and Loebel, 2007). For *in vitro* reconstruction, several groups established protocols to induce definitive endoderm differentiation from human/mouse PSCs, which preferentially differentiate into definitive endoderm in the presence of a high concentration of activin A, which can activate the nodal receptor for activation of the TGFβ signaling pathway. WNT, BMP and FGF signaling also enhanced endoderm specification with activin A (Kubo et al., 2004; Loh et al., 2014; Spence et al., 2011; Yiangou et al., 2018).

#### Definitive endoderm differentiation to the AFE by inhibiting WNT/TGFβ/BMP signaling

After gastrulation, the naïve endodermal sheet is initially divided into the foregut, midgut and hindgut along the AP axis. It is well established that WNT, BMP and FGF signaling promotes hindgut specification but inhibits that of the foregut (Zorn and Wells, 2009). Recapitulating embryogenesis, the blockage of WNT/BMP signaling in PSC-derived definitive endoderm leads to AFE patterning *in vitro*. In addition, Green and colleagues screened growth factors and chemical components that are expected to anteriorly pattern definitive endoderm and found that the inhibition of BMP and TGFβ efficiently induced *SOX2* expression (Green et al., 2011), which may reflect the requirement of TGFβ in AP patterning *in vivo* (Yamamoto et al., 2004). Although the combinations of growth factors and chemicals vary among lab protocols, optimization of this process would obviously increase the efficiency of *NKX2-1* induction (Huang et al., 2014; Longmire et al., 2012; Mou et al., 2012).

#### AFE to NKX2-1^+^ respiratory epithelial progenitors via activation of WNT/BMP/RA signaling

As described above, WNT, BMP and RA are essential growth factors that direct NKX2-1^+^ respiratory epithelial progenitors from the AFE in mouse embryos. Consistently, the combination of WNT/BMP/RA is the minimum requirement necessary for *NKX2-1* induction in human PSCs *in vitro*. FGF2/7/10 and epidermal growth factor are additionally included in several protocols. However, Serra and colleagues described that FGF2 induces thyroid (NKX2-1*^+^*) epithelial cells rather than respiratory epithelium progenitors. Further validation is needed to generate respiratory epithelial progenitors that are more pure (Gotoh et al., 2014; Green et al., 2011; Longmire et al., 2012; Mou et al., 2012; Serra et al., 2017).

#### Trachea-specific epithelial cell markers

As *NKX2-1* is also expressed in the thyroid and neural cells, NKX2-1^+^/PAX8^−^/TUJ1 (TUBB3)^−^ cells have been described as respiratory epithelial progenitors by *in vitro* differentiation (Nikolić et al., 2018). But, this definition is insufficient to separate tracheal progenitors from lung progenitors because both express *NKX2-1* but not *PAX8* or *TUJ1* (Gotoh et al., 2014; Green et al., 2011; Hawkins et al., 2017; Kurmann et al., 2015; Serra et al., 2017). In a recent study, single-cell transcriptome analysis of mouse embryos at E10.5 and E11.5 identified several genes specific to the tracheal epithelium (e.g. *Cldn18*, *Tppp*, *Pcdh10* and *Ly6h*) (Kuwahara et al., 2020). Eenjes and colleagues also reported that *Sox21* is expressed in the epithelium of the mouse trachea but not the lung (Eenjes et al., 2020 preprint). Although the expression of these trachea-specific genes should be validated in human tissues, these genes are potential markers to distinguish the tracheal epithelium from the lung epithelium.

### *In vitro* reconstruction of respiratory mesenchyme toward generating a three-dimensional (3D) trachea

#### PSCs to the middle primitive streak

Mesoderm development begins with the differentiation of epiblasts into the primitive streak. The mesoderm is produced by regionalization of the primitive streak with different potentials along the AP axis. A balance of TGFβ/WNT/BMP signaling coordinates the AP axis of the primitive streak. Reflecting this event, the activation of TGFβ, WNT, BMP and FGF signaling and the inhibition of PI3K signaling recapitulate embryonic development *in vitro* and direct PSCs to the middle primitive streak (Lawson et al., 1991; Loh et al., 2016; Tam and Loebel, 2007).

#### Middle primitive streak to the lateral plate mesoderm

The middle primitive streak can give rise to the paraxial and lateral plate mesoderm (Lawson et al., 1991; Tam and Loebel, 2007). Loh and colleagues successfully guided iPSC-derived primitive streak cells to each mesodermal population by blocking key signaling pathways leading to unwanted cell lineages. In contrast to endoderm differentiation from PSCs, which requires high TGFβ, NODAL and ACTIVIN expression, mesodermal specification is induced by the activation of BMP and the inhibition of TGFβ and WNT (Loh et al., 2014, 2016).

The lateral plate mesoderm can differentiate into several mesoderm derivatives, including the splanchnic mesoderm, cardiac mesoderm and skeletal elements (Prummel et al., 2020). Recent single-cell transcriptomics of developing mouse embryos revealed the dynamics of molecular signatures during this diversification processes, implying that the early splanchnic mesoderm is more exposed to RA than the cardiac mesoderm (Han et al., 2020). Moreover, during *in vitro* reconstruction, the addition of RA to the lateral plate mesoderm decreased the expression of cardiac genes and increased that of splanchnic mesoderm genes. Thus, the activation of RA signaling is needed for efficient tracheal/lung mesenchymal induction from the splanchnic mesoderm.

#### Lateral plate mesoderm to the tracheal/lung mesenchyme

Canonical Wnt signaling ensures the identity of the tracheal mesenchyme by inducing *Tbx4* expression after E10.5 in the mouse (Han et al., 2020; Kishimoto et al., 2020). Based on this finding, we developed a protocol to generate human and mouse tracheal/lung mesenchyme from the induced lateral plate mesoderm. In the mouse, the combined activation of RA, WNT and BMP induced the differentiation of Tbx4^+^ tracheal/lung mesenchyme from the lateral plate mesoderm. By contrast, in the human model, HH (in addition to RA, WNT and BMP) was required for induction of the tracheal/lung mesenchyme (Han et al., 2020; Kishimoto et al., 2020), suggesting that RA, WNT and BMP are evolutionarily conserved inducers, although HH is also required for human tracheal/lung mesenchyme specification. Induced tracheal/lung mesenchymal cells express a set of marker genes including *WNT2* and *TBX4* and can further differentiate into chondrocytes and SMs by extension of the culture duration, demonstrating the potential for proper differentiation to tracheal mesenchyme.

#### 3D modeling of human respiratory tissues using human PSCs

Several laboratories have reported the generation of 3D lung and airway organoids from human PSCs (Chen et al., 2017; Dye et al., 2015; Gotoh et al., 2014; McCauley et al., 2018, 2017; McCracken et al., 2014). In a recent study using tracheal xenograft implantation, human PSC-derived basal cells formed an airway epithelium similar in structure and composition to *in vivo* airways (Hawkins et al., 2021). However, the generation of tracheal organoids containing mature airway epithelial cells surrounded by cartilage and SM remains a challenge. In 3D lung organoids, the mesenchyme co-develops with NKX2-1^+^ respiratory epithelium progenitors, indicating the need for synchronized development of the epithelium and mesenchyme. Dye and colleagues first reported the generation of lung organoids from human PSCs by the combination of a recapitulating developmental process and spontaneous spheroid formation technique (Dye et al., 2015; Spence et al., 2011). Long-term culture by transplantation into the mouse kidney capsule matures the tissue structure and cell composition. Transplanted organoids exhibited cartilage nodules and *SOX9* expression, as well as SM structure (Dye et al., 2016). More recently, Chen and colleagues generated lung bud organoids with mesenchyme expressing pulmonary markers, including *TBX4* and *HOX5* paralogs (Chen et al., 2017).

Although it remains unclear whether these organoids contain tracheal cells, the presence of proximal epithelial cells [p63 (TP63)^+^ basal cells and goblet cells] and cartilage implies the presence of tracheal cells. Further characterization of cell types in these organoids with single-cell RNA sequencing would enable us to distinguish the trachea and lungs. The generation of pure tracheal organoids remains challenging because we are just beginning to understand what is unique and common between the trachea and lungs. Although further validation with region-specific marker genes is needed to distinguish trachea and lung tissue, lung organoids are promising platforms to generate tracheal organoids by, for example, more anterior patterning of these organoids.

## Conclusions

Over the past few decades, cumulative genetic and genomic studies using animal models have expanded our knowledge of tracheal/lung development, which has enabled scientists to mimic the developmental processes of tracheal/lung tissues from human PSCs in a dish. However, as our knowledge of developmental principles have been largely based on genetic studies and biased to gene regulatory networks and cell signaling pathways, the behaviors of living cells and mechanical regulation are poorly understood. Furthermore, studies of human organogenesis remain challenging because of the inaccessibility of human fetal tissues and ethical concerns. The generation of tractable organoid models that can be manipulated will help in overcoming the difficulty in studying human trachea development. 3D organoid culture technologies have already proven immensely useful for understanding human development and regeneration, but current models are limited by the immature structure and physiology (Takebe and Wells, 2019). For example, despite the large size of the human trachea, lab-grown organoids are still too small and are spherical, rather than tubular. Although current organoid models can be used to develop diverse cell types and tissues, these lack structural complexities, such as oriented SM and periodically patterned cartilaginous rings. Additionally, the induction of blood vessels and nerves would improve tissue maturation and tissue shaping (Grebenyuk and Ranga, 2019; Workman et al., 2017). The self-organization potential of stem cells may be insufficient to generate an implantable artificial trachea with an accurate tube structure. Tissue engineering could help generate more authentic tubular tracheal tissues. For example, 3D bioprinting technologies would aid in building an implantable artificial trachea; however, there are still some difficulties that remain, such as optimizing scaffold materials and cell sources.

Another problem is the distinction between the trachea and lung. Although some studies have identified markers specific to the tracheal and lung epithelium, we are only now beginning to understand the differences. Further exploration of region-specific marker genes, especially in the mesenchyme, is necessary for the generation of pure tracheal organoids. Rapidly expanding single-cell RNA sequence technologies will help in identifying lineage-specific marker genes to determine individual cell types.

Although the generation of authentic tracheal organoids is underway, future analyses of tracheal organoids with emergent transcriptome and live-imaging techniques at single-cell resolution as well as tissue-engineering technologies would open an unprecedented window in the study of human tracheal development.
